# Simultaneous Recordings of Human Microsaccades and Drifts with a Contemporary Video Eye Tracker and the Search Coil Technique

**DOI:** 10.1371/journal.pone.0128428

**Published:** 2015-06-02

**Authors:** Michael B. McCamy, Jorge Otero-Millan, R. John Leigh, Susan A. King, Rosalyn M. Schneider, Stephen L. Macknik, Susana Martinez-Conde

**Affiliations:** 1 Barrow Neurological Institute, Phoenix, Arizona, United States of America; 2 Department of Signal Theory and Communications, University of Vigo, Vigo, Spain; 3 Department of Neurology, Johns Hopkins University, Baltimore, Maryland, United States of America; 4 Veterans Affairs Medical Center, Case Western Reserve University, Cleveland, Ohio, United States of America; 5 SUNY Downstate Medical Center, Brooklyn, New York, United States of America; University of Leicester, UNITED KINGDOM

## Abstract

Human eyes move continuously, even during visual fixation. These “fixational eye movements” (FEMs) include microsaccades, intersaccadic drift and oculomotor tremor. Research in human FEMs has grown considerably in the last decade, facilitated by the manufacture of noninvasive, high-resolution/speed video-oculography eye trackers. Due to the small magnitude of FEMs, obtaining reliable data can be challenging, however, and depends critically on the sensitivity and precision of the eye tracking system. Yet, no study has conducted an in-depth comparison of human FEM recordings obtained with the search coil (considered the gold standard for measuring microsaccades and drift) and with contemporary, state-of-the art video trackers. Here we measured human microsaccades and drift simultaneously with the search coil and a popular state-of-the-art video tracker. We found that 95% of microsaccades detected with the search coil were also detected with the video tracker, and 95% of microsaccades detected with video tracking were also detected with the search coil, indicating substantial agreement between the two systems. Peak/mean velocities and main sequence slopes of microsaccades detected with video tracking were significantly higher than those of the same microsaccades detected with the search coil, however. Ocular drift was significantly correlated between the two systems, but drift speeds were higher with video tracking than with the search coil. Overall, our combined results suggest that contemporary video tracking now approaches the search coil for measuring FEMs.

## Introduction

Gaze position can indicate the locus of attention and provide information about visual processing strategies [1–5]. Measurement of pathological eye movements can also help understand the pathogenesis of neural disease [6–9]. The accurate measurement of eye movements is important to numerous research areas, including visual neuroscience, scene perception [1,10], machine vision [11], clinical research [6,12–15], and other applied fields [4,16–19], but it is perhaps most critical to studies on fixational eye movements (FEMs; the involuntary, small-magnitude eye movements continuously produced during fixation: i.e. microsaccades, drift and tremor), due to the eye tracking sensitivity and precision required to obtain reliable FEM data.

Researchers have investigated FEMs for over 50 years [2,20–24]. Human studies from the 1950s relied on a contact lens-based method, called the "optical lever" method, to track eye movements. This involved imbedding one or two reflecting mirrors in a contact lens, placing the contact lens on the participant’s eye, and then shining a light source on it and recording the reflected light, which led to a signal about the orientation of the eye [21]. The optical lever method went out of use, due its complexity and invasiveness, with the arrival of the search coil method in the 1960–1970s [25,26]. The search coil method involves placing a contact lens, containing coils of thin copper wire, on the eye of a participant who sits inside an area including magnetic fields that induce gaze-dependent electric currents in the coils (search coils are surgically implanted in animal models). The induced current signals travel through a thin wire that leaves the corner of the eye. The search coil is considered the gold standard for eye tracking, being significantly less noisy than video trackers [27–32], but it is invasive and may interfere with natural vision. Additional pitfalls are limited experimental time due to the subject's discomfort, necessary use of topical anesthesia, potential drying of the eye from inadequate blinking, temporary deformations of the cornea, and reduced visual acuity [29]. Further, saccades may be slowed when measured by search coils [31,33], although recent evidence argues against this hypothesis [34]. Some recent FEM studies have used dual Purkinje eye image (DPI) trackers, which track the fourth Purkinje image and the corneal reflection [35], but these typically require the use of a bite bar as well as an eye patch (since recordings are almost always monocular), in addition to a bulky and complicated setup [36]. Because of these difficulties, most contemporary researchers rely on video-oculography (i.e. video tracking) methods to record human eye movements—including FEMs—, as they are easiest to use, most flexible, and interfere with natural vision the least. Video tracking methods use a camera to digitally film landmarks on the eye (sometimes in conjunction with recording optical reflections from the eye), to determine the eye's orientation. The most common video tracking method tracks the pupil (with or without the corneal reflection).

As stated earlier, the reliable measurement of microscopic eye movements (i.e. microsaccades and ocular drift (hereafter drift)) is critical to FEM research, but no studies have conducted an in-depth comparison of human FEM recordings obtained with the search coil method (the gold standard for measuring FEMs) versus those obtained with contemporary, state-of-the art video trackers.

Previous human studies compared search coil recordings with a variety of video tracking recordings, but they did not investigate FEMs [27,28,30–33,37,38]. Recent research [34] pioneered simultaneous recordings with the scleral search coil technique and the most popular video tracking system in current FEM research (EyeLink 1000, SR Research) in head-fixed primates (i.e. stabilized with a surgical headpost). This study included an analysis of microsaccade detection with both systems (i.e. a comparison of the magnitude distributions of microsaccades detected with both systems versus those detected with one system exclusively), as well as an analysis of the effect of pupil size changes (caused by luminance changes) on (erroneous) gaze position, but did not investigate the properties of corresponding microsaccades in the two systems, or compare drift between the two systems.

It is also worth noting that human experimental setups typically stabilize the head with a head and/or chinrest, thereby allowing substantially more head motion than in headpost-implanted primates. In addition, whereas in non-human primates search coils are surgically implanted in the sclera of the eye, in human subjects they are embedded in a contact lens that is rested on top of the eye. The differential effects (or lack thereof) of both techniques on eye movement recordings have been debated [25,33,34]. Thus, the primate results in [34] may or may not generalize to most psychophysical and clinical human studies conducted with contact lens-mounted search coils and standard head- and/or chin-rests, especially for research concerning FEMs.

Here we provide an in-depth study of human FEMs (microsaccades and drift) measured simultaneously with a search coil and a state-of-the-art video tracker (EyeLink 1000, SR Research). One important feature of the present study is that it was conducted in experimental conditions that are characteristic of most human research on FEMs (whereas implanted headposts such as those in [34] immobilize the head to a larger degree than the chin/forehead rests generally used in human studies).

Participants fixated a small target while we recorded their eye movements simultaneously with a search coil and video tracker. We found substantial agreement between the two recording systems, but also some differences. 95% of microsaccades detected with the search coil were also detected with the video tracker, and 95% of microsaccades detected with video tracking were also detected with the search coil. This minor disagreement in microsaccade detection was largely constrained to microsaccades smaller than 0.5 degrees of visual angle. Further, the peak/mean velocities and main sequence slopes of microsaccades detected with video tracking were significantly higher than their search coil counterparts. Horizontal and vertical eye positions during ocular drift were significantly correlated between the two recording systems, but drift speeds were higher with video tracking than with the search coil method.

## Materials and Methods

### Participants

Five subjects (3 males, 2 females) participated in the experiment. All of them had healthy eyes (normal or corrected-to-normal vision) and no visual complaints when they wore their refractive corrections. The subjects did not wear eye glasses or corrective lenses during the experiment, but had no difficulties seeing, or fixating their gaze on, the fixation target. No subjects experienced previous corneal surgery. These experiments were performed in approval of the Cleveland Veterans Affairs Medical Center Institutional Review Board and in conformity with the Declaration of Helsinki. All participants gave written informed consent.

### Experimental design

Before starting the experiment, a licensed physician applied a drop of topical anesthetic to the eye that was to have the contact lens-mounted search coil. The contact lens was then placed in the participant’s eye.

Participants sat in a custom-designed chair with head support 1.2 m away from a tangent screen, and fixated their gaze on a small red laser spot presented at the center of their field of view. The experiment started with the five-point calibration procedure built into EyeLink 1000 (center, 5 deg up, 5 deg down, 5 deg left, 5 deg right; this was later used to calibrate each set of data offline), and consisted of 2 trials of one minute each.

### Eye movement recordings

#### Video tracker

We used the EyeLink 1000 eye tracking system (SR Research) to obtain binocular eye position from participants 1, 3, 4, and 5 and monocular eye position (right eye) from participant 2 (we could not obtain binocular eye position for this subject to a technical difficulty with the eye tracker data). We note that, even though binocular eye position was available from 4 subjects, we only compared eye movement data collected from the same eye in both recording systems (video tracking and search coil method). We did this to avoid potential confounding factors when interpreting differences between the two systems; for instance, the search coil may influence saccadic metrics [39] and oculomotor adaptation due to the load of the search coil may [40–42] or may not [41,43] transfer to the eye without the search coil. Further, because drifts are not generally conjugate [24,44,45], comparisons between the two systems must be made on the same eye. The EyeLink system accounts for minor head movements using the corneal reflection. Thus, we obtained head-referenced eye-rotation angles by tracking the pupil and corneal reflection with the following video tracker settings: we set the pupil tracking method to centroid, turned off the heuristic filter, and used the built-in auto threshold. Participant 3 was discarded due to high noise levels in the video tracker data, and one trial was discarded for participant 1 because of technical difficulties.

#### Search coil

We recorded eye position monocularly for each subject (right eye for participants 1, 2, and 3; left eye for participants 4 and 5). Horizontal and vertical eye movements were measured with 1.83 m field coils (CNC Engineering, Seattle, WA) that used a rotating magnetic field in the horizontal plane and an alternating magnetic field in the vertical plane. We put a head coil on to measure head rotations. Thus, the eye-in-head rotations are the difference between the eye (gaze) coil signal and the head coil signals. Search coils were calibrated before each experimental session using a protractor device (this was in addition to the post hoc calibration performed offline and described next).

#### Sampling, calibration, synchronization, and post processing of search coil and video tracker data

The raw analog output of EyeLink was sampled and recorded using the search coil system at 500 Hz. This allowed perfect synchronization of the search coil and video tracker data. The video tracker and search coil data received the same processing (post and online); thus differences between the data sets are not due to differences in processing methods.

To avoid aliasing, the analog search coil signals (and also the analog EyeLink signals) were passed through Krohn—Hite Butterworth filters (bandwidth 0–150 Hz) before digitization at 500 Hz with 16-bit resolution. The digitized search coil and EyeLink signals were then filtered with an 80-point software filter (Remez FIR; bandwidth 0–100 Hz).

The raw signals from both sets of recorded data were calibrated offline using the data from the five-point calibration procedure built into EyeLink 1000. To do this, we performed robust linear fits (using Matlab’s built in robustfit function) for each data set. Thus, each system was calibrated the same way and any differences between the data sets are not due to differences in calibration.

**Fig 1:**
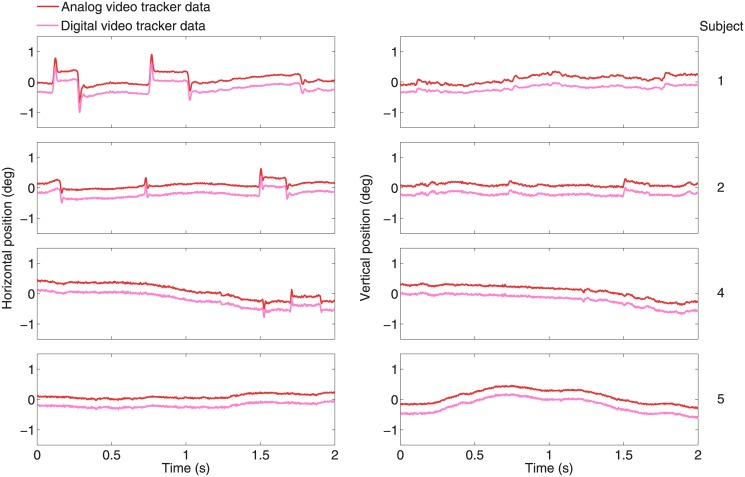
Comparison of data recorded from the analog versus the digital output of the video tracker. A two second epoch of analog versus digital eye movement recordings for each subject (indicated at the right) for the horizontal (left) and vertical (right) eye positions. Red traces are the digital signals obtained from the analog output of the video tracker as described in the Methods section. Pink traces are the corresponding data obtained from the digital output of the video tracker. Both signals are nearly identical.

A word of caution is necessary concerning analog recordings with EyeLink: analog data transfer may substantially degrade data quality compared to digital transfer via the link built into the EyeLink [46]. To ensure that analog data transfer did not worsen our data quality significantly, we also recorded the digital signal (calibrated with the same 5-point procedure as the analog signal) and compared it to the digital signal after conversion from the analog output (Fig 1). We calculated the Pearson correlation coefficient (calling the Matlab routine "corr") and found, on average across subjects, a mean correlation in the horizontal component of 0.996, and in the vertical component of 0.993. Thus our analog-to-digital conversion introduced, at most, negligible amounts of noise.

### Eye movement analyses

#### Blinks, microsaccades, and overshoots

**Fig 2:**
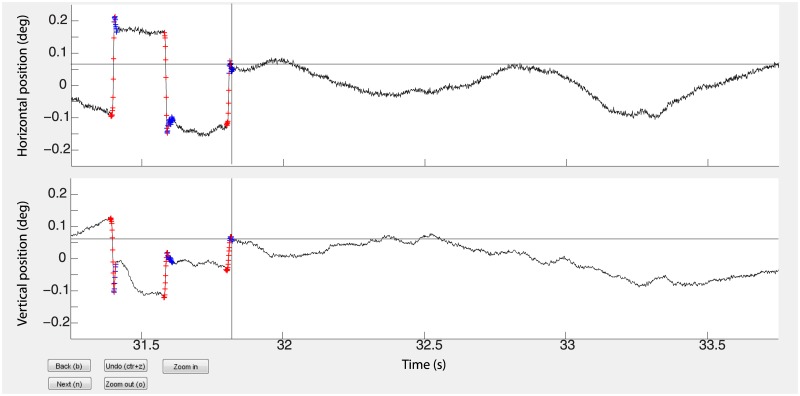
Microsaccade and overshoot detection. Here we show the horizontal (top black trace) and vertical (bottom black trace) EyeLink data from subject 5 over a 2.5 second epoch of time. Microsaccades were manually detected by aligning (using the mouse as control) the cross hairs, shown simultaneously on the horizontal and vertical axes, with the start of the microsaccade and clicking the mouse, and then aligning to the stop of the microsaccade and clicking the mouse. Overshoots were detected in the same way. An overshoot always began at the sample after the end of the microsaccade. The red crosses on the black traces indicate the defining samples of microsaccades and the blue crosses indicate the defining samples of the overshoots.

Some saccades are followed by a fast small saccadic, oppositely directed, eye movement called overshoot, which is often more prominent for the eye that moves in the abducting direction [47]. Unlike the return saccade in a square-wave jerk, a dynamic overshoot follows a saccade without latency between the two movements. Blinks, microsaccades, and overshoots were manually tagged in both data sets by an experienced investigator (author M.B.M.), by delineating the start and stop sample points of each event using a custom Matlab program (Fig 2). We note that the expert was not blind to the source of the data (video vs. search coil). However, whenever he was coding data from one system, he was blind to the data from the other system. This was to avoid potential bias in detecting or rejecting a saccade based on its presence/absence in the other system.

**Fig 3:**
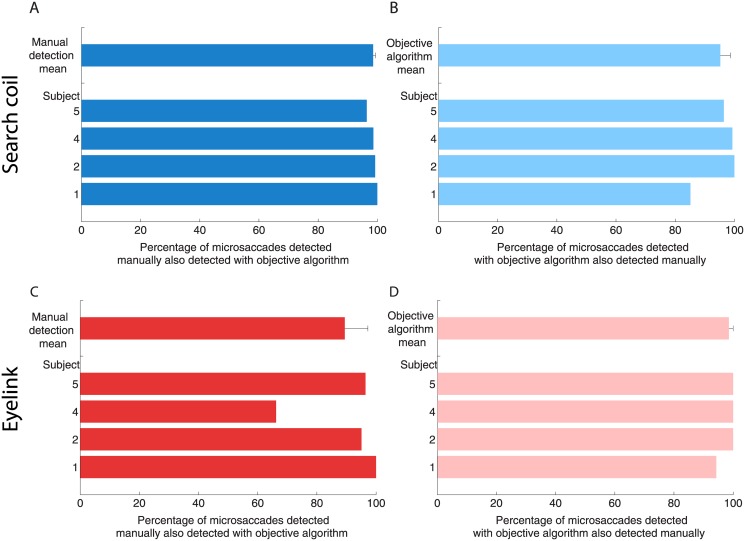
Coherence of detection between manually detected microsaccades and microsaccades detected with an objective algorithm, for the search coil (top row) and video tracker (bottom row) data. (**A**) Percentage of search coil microsaccades detected manually that were also detected with the objective algorithm. (**B**) Percentage of search coil microsaccades detected manually that were also detected with the objective algorithm. (**A**) Percentage of video tracker microsaccades detected manually that were also detected with the objective algorithm. (**B**) Percentage of video tracker microsaccades detected manually that were also detected with the objective algorithm. Error bars represent the S.E.M. across participants (*n* = 4).

We displayed the horizontal and vertical eye positions (on separate vertically aligned axes). A cross hair, targeting the eye position, was displayed on each axis and could be moved with high precision control (at the temporal resolution of the sample rate, i.e. 500 Hz) with the mouse, allowing the experienced investigator to select and click (with the mouse) on the start sample and subsequently select and click on the stop sample. If a microsaccade was followed by an overshoot, the duration of the microsaccade was later refined to include the overshoot. Manual detection precluded the potential confound that any observed differences between data sets could be due to differences in the performance of an objective algorithm. For instance, the presence of many false alarms/negatives in one dataset (i.e. subject 1 in Fig 3B and subject 4 in Fig 3C) could lead to the conclusion that one system performed better than the other, whereas the discrepancy would have been due to the algorithm. Manual tagging also allowed us to delineate very precisely the stops and starts of blinks, microsaccades, and overshoots; this was especially critical to the analyses concerning overshoots. The average number of microsaccades detected per subject was 116.75 (± 29.62 s.e.m) for the video tracker and 116.25 (± 29.54 s.e.m.) for the search coil.

Notwithstanding the above, we found comparable results about the properties of microsaccades, overshoots and drifts when using manual detection and when applying the objective algorithm developed by Engbert and Kliegl [48] (Fig 3). On average, 98.6% of the search coil microsaccades detected manually were also classified as microsaccades using the objective algorithm, and 95.2% of the search coil microsaccades detected objectively were also classified as microsaccades manually (Fig 3A and 3B). On average, 89.4% of the video tracker microsaccades detected manually were also classified as microsaccades using the objective algorithm, and 98.6% of the video tracker microsaccades detected objectively were also classified as microsaccades manually (Fig 3C and 3D). All analyses, unless otherwise specified, were performed on manually detected microsaccades and overshoots.

#### Microsaccade detection with an objective algorithm

We also detected saccades with a modified version of the algorithm developed by Engbert and Kliegl [48–52] with λ = 6 and a minimum saccadic duration of 6 ms. We identified dynamic overshoots as saccades that occurred less than 20 ms after a preceding saccade [53] and we did not regard them as new saccades. Instead, we added the duration of the overshoot into the duration of the saccade, thus considering it part of the saccade. Microsaccades were defined as saccades with magnitude < 2 deg in each eye [54–58].

**Fig 4:**
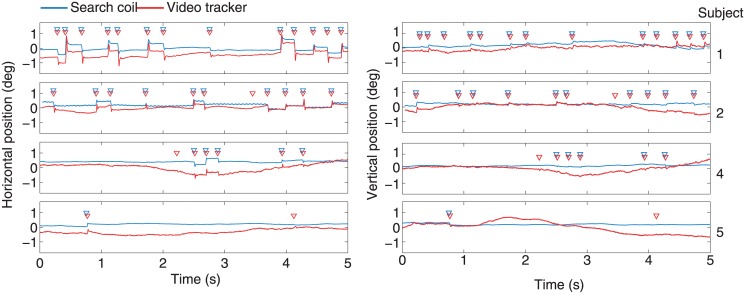
Examples of simultaneous horizontal and vertical eye position traces obtained with the search coil and video tracking, for each participant. Red triangles represent microsaccades detected with the video tracker; blue triangles represent microsaccades detected with the search coil. Note that microsaccades were detected by viewing the vertical and horizontal traces simultaneously. For illustration purposes, a small offset of 0.2 deg has been added to the search coil data.

The most popular contemporary microsaccade-detecting algorithm used here, and developed by Engbert and Kliegl [48], relies on a velocity threshold, determined by the parameter λ (the number of standard deviations above median velocity in a trial to be considered above the velocity threshold), that adapts to the level of noise in a given recording. Because video tracker data is typically nosier than search coil data (mean RMS for this experiment: video tracker 7.1 ± 1.1 deg/s, search coil 4.0 ± 0.6 deg/s), velocity thresholds for video tracker data are usually higher than for search coil data (mean polar-velocity thresholds for this experiment: video tracker 10.5 ± 0.8 deg/s, search coil 5.0 ± 0.6 deg/s). This complicates data interpretation, because differences in detection might be due to differing levels of noise, differing velocity thresholds, or both. For instance, we found that video tracker microsaccades had statistically significant shorter durations than their search coil counterparts when using the objective algorithm to detect microsaccades (data not shown). This is most likely due to the differential performance, on each recording system's data, of the microsaccade detection algorithm (as evidenced by the equal microsaccade durations between the two systems when using manual detection): higher noise levels in the video tracker data may have reduced the time during which eye movement speeds were above threshold, thereby shortening microsaccade durations, and/or lower noise levels in the search coil data may have increased the time during which eye movement speeds were above threshold, thus lengthening microsaccade durations. To avoid such confounds, we used manual detection of microsaccades for all the main analyses (triangles in Fig 4 point to manually detected microsaccades).

#### Microsaccade properties

**Fig 5:**
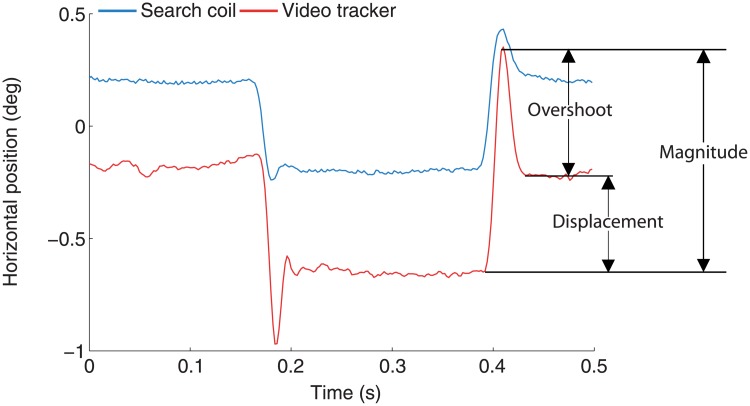
Horizontal components from the vectors used to calculate microsaccade magnitude, displacement, and overshoot size (see Methods for the precise definitions of these terms). The vertical components of the vectors were defined similarly. Arrows indicate the magnitude, displacement, and overshoot of a video-tracked microsaccade. For illustration purposes, a small offset of 0.2 deg has been added to the search coil data.

We defined microsaccade magnitude as the magnitude of the 2-D vector resulting from the largest possible horizontal and vertical vectors made up from the samples comprising the microsaccade. Overshoot magnitude was defined similarly, as the magnitude of the 2-D vector resulting from the largest possible horizontal and vertical vectors made up from the samples comprising the overshoot. We defined microsaccade displacement as the magnitude of the vector from the beginning sample to the end sample of the microsaccade (Fig 5). We note that magnitude, overshoot and displacement are not independent measures (i.e. because magnitude is the sum of overshoot and displacement). We have chosen to include all three measures, despite their interdependency, to facilitate data comparison with other studies (where specific definitions of microsaccade magnitude may be lacking).

All microsaccade and overshoot properties were calculated from the raw data using custom Matlab code. The microsaccade main sequence slopes were calculated by fitting a line to the peak-velocity/magnitude relationship by total least-squares (i.e. errors were assumed to be in the independent and dependent variables).

**Fig 6:**
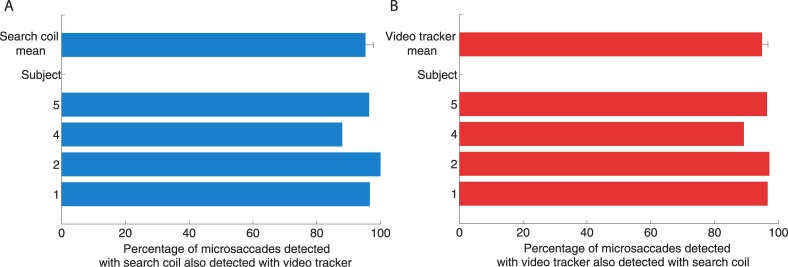
Coherence of detection between the two recording systems. (**A**) Percentage of microsaccades detected with the search coil that were also detected with the video tracker. (**B**) Percentage of microsaccades detected with the video tracker that were also detected with the search coil. Error bars represent the S.E.M. across participants (*n* = 4).

**Fig 7:**
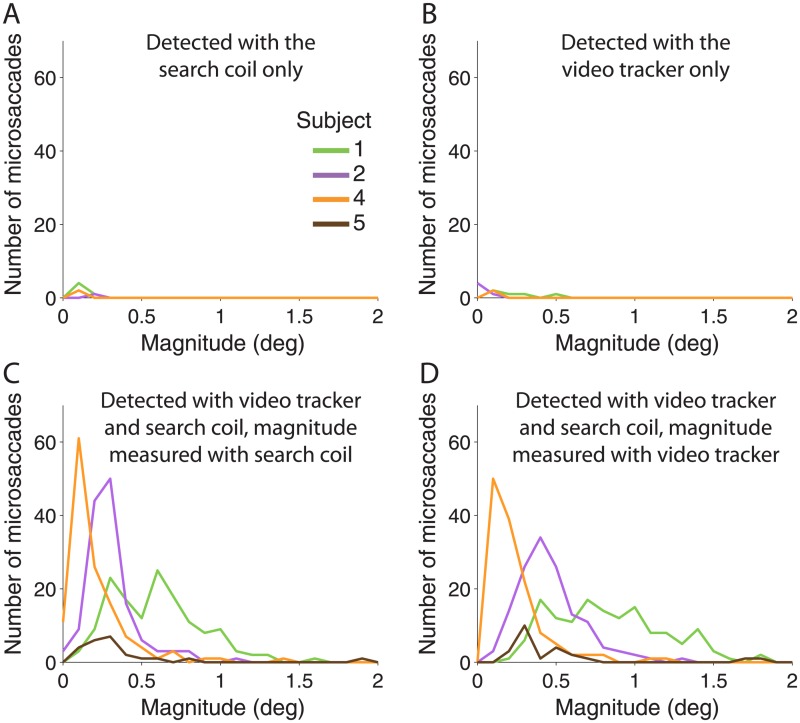
Magnitude distributions for microsaccades detected in only one recording system or in both systems. Magnitude distributions of microsaccades detected in only one system (**A–B**) or both systems (**C–D**). Detection system indicated at the top of each panel.

**Fig 8:**
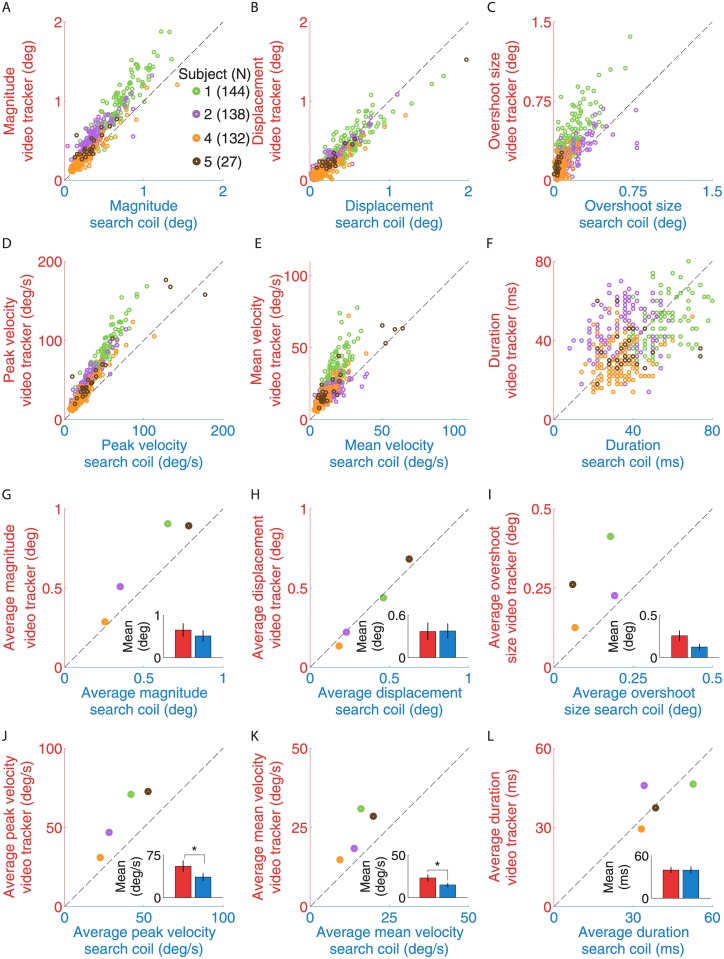
Comparison of microsaccade properties for microsaccades detected in both recording systems. Scatter plots for microsaccade magnitude (**A**), displacement (**B**), overshoot size (**C**), peak velocity (**D**), mean velocity (**E**), and duration (**F**). Each dot in (**A–F**) represents one microsaccade, with the property as measured with the video tracker on the *y*-axis and with the search coil on the *x*-axis. Average microsaccade magnitude (**G**), displacement (**H**), overshoot size (**I**), peak velocity (**J**), mean velocity (**K**), and duration (**L**), for each participant (i.e. average data from panels **A–F**). Each dot represents the average microsaccade property for one participant as measured with the video tracker on the *y*-axis and with the search coil on the *x*-axis. In the legend in panel (**A**), N indicates the number of common microsaccades detected in both data sets. Bar insets in (**G–L**) are the video tracker (red) and search coil (blue) averages across participants. Error bars represent the S.E.M. across participants (*n* = 4). * indicates statistical significance with a *p*-value < 0.05 (two-tailed paired *t*-tests).

We considered a video tracker microsaccade and a search coil microsaccade to be one and the same if they had any time overlap in both recording systems (Figs 6–8). We considered a video tracker overshoot and a search coil overshoot to be one and the same if they belonged to corresponding microsaccades.

#### Ocular drift

**Fig 9:**
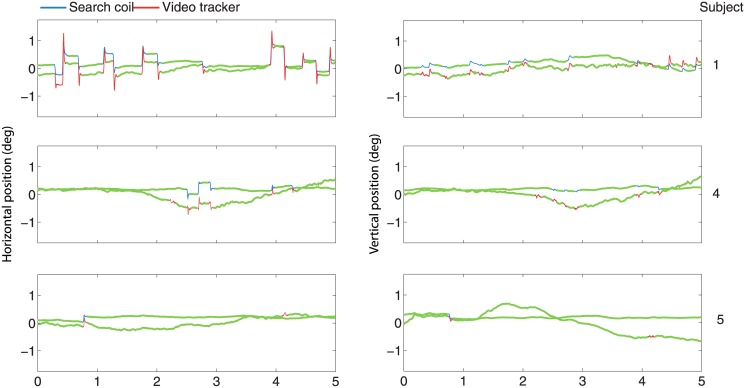
Examples of simultaneous horizontal drift traces (green lines) obtained with the search coil and video tracker, for each participant contributing to the drift analyses. For illustration purposes, a small offset of 0.2 deg has been added to the search coil data.

Drift periods were defined as the eye-position epochs between microsaccades, overshoots, and blinks. We removed 10 ms from the start and end of each drift period (because of imperfect detection of blinks and microsaccades), and filtered the remaining eye-position data with a low-pass Butterworth filter of order 13 and a cut-off frequency of 30 Hz [59]. We removed an additional 10 ms from the beginning and end of each drift period to reduce edge effects due to the filter. Drifts shorter than 50 ms were discarded. Note that the low pass filter cannot completely remove all noise; thus, because video tracker data is typically noisier than search coil data, it is possible for noise to remain higher, post-filter, in the video tracker data than in the search coil data. We compared the horizontal and vertical eye positions during ocular drift (green traces in Fig 9) and instantaneous speeds (i.e. absolute values of the instantaneous velocity; derivative of green traces in Fig 9) of drift segments occurring over identical periods of time in the two systems, using the filtered data (green traces in Fig 9; see Statistical analyses below), which has the same temporal resolution (i.e. 500 Hz) as the original data. In addition, we compared drift displacements and distances across the two systems. The displacement was defined as the magnitude of the 2D vector joining the eye position at the beginning of the drift and the eye position at the end of the drift and the distance was defined as the length of the curve traced out by the eye position in 2D space over the duration of the drift. We calculated each drift property (speed, displacement, distance) for each drift epoch and then averaged across epochs for each subject. Oscillatory noise in the search coil data precluded drift analyses for participant 2 (Fig 4, blue trace of horizontal component).

### Statistical analyses

We calculated correlations, across both eye tracking systems, for the 2D components (i.e. horizontal and the vertical) of eye position during ocular drift. To compare horizontal ocular drift between the two systems, we calculated Spearman's correlation coefficient (*ρ*) between all the eye position data samples (green traces in Fig 9) declared as drift in both data sets (Table 1) (this was similarly done for vertical ocular drift), for each subject.

**Table 1:** Drift descriptive statistics and correlations between ocular drift measured with the video tracker and ocular drift measured with the search coil.

Drift descriptive statistics			Horizontal ocular drift correlations		Vertical ocular drift correlations	
Subj.	Num.	Avg. duration (ms)	*ρ*	*p*-value	*ρ*	*p*-value
1	128	271	0.68	< 10^−30^	0.58	< 10^−30^
4	159	614	0.47	< 10^−30^	0.74	< 10^−30^
5	33	3449	0.37	< 10^−30^	0.43	< 10^−30^

The three columns on the left side of the table indicate the subject and descriptive statistics for the given subject. The four columns on the right are the Spearman correlation coefficients (*ρ*) and the *p*-values obtained for each correlation in the horizontal and vertical component.

We also compared eye position during ocular drift and absolute pupil size by calculating Spearman's correlation coefficient between the eye position during ocular drift and the absolute pupil size in the video tracker data. To do this, we measured the absolute value of the pupil size over the duration of the experimental session, and correlated all pupil size samples with the corresponding ocular drift samples.

We used two-tailed paired *t*-tests for all other comparisons. All *t*-tests were done across subjects, rather than across saccades or drifts. The significance level was set to α = 0.05 throughout.

## Results

Human participants fixated a central target with their head supported while we recorded their eye positions simultaneously with a search coil and a fast video tracker (EyeLink 1000, SR Research). An experienced investigator (author M.B.M.) manually identified blinks, microsaccades, and overshoots from each recording system's data. Drift was defined as the eye movements occurring between microsaccades and blinks (see Methods for details). We then determined the agreement in microsaccade detection between the two systems, as well as the properties of microsaccades and drift measured using the two systems. Because all post and online processing procedures were equivalent for both data sets, any differences reported are due to differences in the recording systems.

### Coherence of detection

There was good qualitative correspondence between the microsaccades detected with each system, for each participant (Fig 4). The RMS was higher for the EyeLink data than the search coil data, however, suggesting higher levels of noise (mean RMS for this experiment: video tracker 7.1 ± 1.1 deg/s, search coil 4.0 ± 0.6 deg/s). To quantify any differences between the two systems, we first compared the coherence of microsaccade detection between the two systems. To do this, we declared a microsaccade detected with the video tracker and a microsaccade detected with the search coil to be one and the same if the two had any overlap in time. On average, 95 ± 3% of the search coil microsaccades were also classified as video tracker microsaccades, and 95 ± 2% of the video tracker microsaccades were also classified as search coil microsaccades (Fig 6).

Thus, our results indicate comparable microsaccade detection for video tracking and search coil data. Yet, 5% of microsaccades classified in one system were not classified in the other. This minor disagreement could be due to noise or errors in tagging by the experienced investigator (author M.B.M.). Upon inspecting the magnitude distributions of common and mutually exclusive microsaccades, we found that the discrepancy was largely constrained to microsaccades smaller than 0.5 deg (Fig 7); 89% of microsaccades smaller than 0.5 degrees detected with the search coil were also detected with the video tracker, and 92% of microsaccades smaller than 0.5 detected with video tracking were also detected with the search coil method. At the other end, 100% of microsaccades larger than 0.5 degrees detected with the search coil were also detected with the video tracker, and 99.8% of microsaccades larger than 0.5 degrees detected with video tracking were also detected with the search coil method. We next quantify any differences in the properties of microsaccades detected with both systems.

### Coherence of microsaccade properties between search coil and video tracker recordings

To determine whether the properties of those microsaccades detected with both recording systems might differ, we measured each property using the same method for each dataset. We detected an average of 110 microsaccades per subject. We found that the peak/mean velocity of microsaccades detected with the video tracker was significantly larger than in their search coil counterparts (Fig 8). Microsaccade magnitude, displacement, overshoot size, and duration were not significantly different between the two systems (Fig 8). Finally, main sequence slopes for the peak-velocity/magnitude relationship were significantly higher for the video tracker than for the search coil (search coil: 75 ± 4 s^-1^; video tracker: 102 ± 5 s^-1^; *t*(3) = 4.12, *p* = 0.025). These results indicate that video tracker and search coil measurements of microsaccade properties differ in some ways.

### Ocular drift

#### Coherence of drift properties between search coil and video tracker recordings

We compared the horizontal and vertical ocular drift (green traces in Fig 9) between the video tracker data and the corresponding search coil data, for 3 participants (see Methods for details). Correlations between video tracker drifts and their search coil counterparts were moderate (average vertical *ρ* = 0.58 ± 0.09; average horizontal *ρ* = 0.51 ± .09) but highly significant (all *p*-values < 10^–30^). We also found that 2D video tracker drift speeds were significantly higher than those measured with the search coil (average 2D drift speed: search coil 0.56 ± 0.19 deg/s; video tracker 1.24 ± 0.10 deg/s; *t*(2) = 7.01, *p* = 0.02). Whereas the ocular drift speed was higher for the video tracker data than for the search coil data, neither the 2D displacement (average displacement: search coil 0.10 ± 0.01 deg/s; video tracker 0.38 ± 0.17 deg/s; *t*(2) = 1.74, *p* = 0.22) nor the 2D distance traversed by the eye during the drift (average distance: search coil 0.47 ± 0.24 deg/s; video tracker 1.50 ± 0.93 deg/s; *t*(2) = 1.48, *p* = 0.27) differed significantly between the two systems.

#### Drift and pupil size interactions

**Fig 10:**
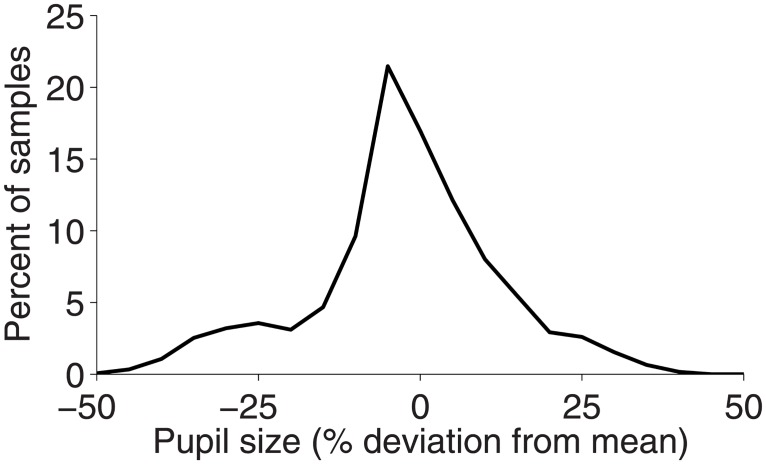
Pupil size fluctuations. Distribution of pupil size deviations from the mean pupil size for all subjects considered in the drift analyses (*n* = 3).

Pupil size fluctuations can cause erroneous reports of eye position changes in pupil-based video trackers [34,38]. Absolute pupil size (heretofore, pupil size) did not fluctuate much in the present study, as 75% of all pupil size samples were within 15% from the mean (Fig 10), but we did find a correlation between pupil size and ocular drift measured with the video tracker (Table 2). It is difficult to fully unravel the implications of this result on the correlations between ocular drift across the two systems, because pupil size fluctuations can be due to eye movements changing the pupil size from the perspective of the video tracker camera (i.e. actual eye movements), or to fluctuations in luminance, arousal, or other factors. Most plausibly, the correlation between pupil size and ocular drift in the video tracker may explain why we did not find higher correlations between ocular drift across the two systems. That is, whereas pupil fluctuations not associated with changes in gaze position will produce erroneous drift in the video tracker, the search coil will only measure true rotations of the eye. Future research may validate or disprove this possibility by conducting simultaneous search coil and video recordings, while changing luminance levels in a controlled manner (i.e. as in [34,38]). Whereas prior studies did not analyze pupil size fluctuations in relationship to ocular drift per se ([34,38]) they did suggest, as the present research does, that fluctuations in pupil size will cause erroneous ocular drift measurements. Thus, previous findings [34,38], combined with the current results, indicate that care must be taken when using a video tracker to measure ocular drift. In particular, one must carefully control variables such as luminance, arousal, or any others known to influence pupil size, for proper conclusions to be reached.

**Table 2:** Drift and pupil size.

Correlation of horizontal ocular drift with pupil size			Correlation of vertical ocular drift with pupil size	
Subj.	*ρ*	*p*-value	*ρ*	*p*-value
1	-0.21	< 10^−30^	-0.28	< 10^−30^
4	-0.23	< 10^−30^	-0.64	< 10^−30^
5	0.36	< 10^−30^	0.35	< 10^−30^

Correlations of pupil size with horizontal and vertical ocular drift.

## Discussion

Human subjects fixated a central spot while we recorded their eye movements simultaneously with a fast video tracker (EyeLink 1000) and a search coil system. Our results indicate substantial agreement between the microsaccades and drift measured with each recording system. On average, 95% of microsaccades detected with the search coil were also detected with the video tracker, and 95% of microsaccades detected with the video tracker were also detected with the search coil. Microsaccade and drift properties measured with each system differed in some ways, however. Video tracker microsaccade magnitudes, peak/mean velocities, overshoot sizes, and main sequence slopes were significantly higher than their search coil counterparts. Horizontal and vertical ocular drift were significantly correlated between the two systems, but drift speeds were significantly larger for video tracker than for search coil data. We discuss possible explanations for our combined findings next.

### Differences in microsaccade detection and properties

The small disagreement in microsaccade detection across the two systems could be due to noise and/or classification errors by the experienced investigator tagging the microsaccades (author M.B.M.). The larger peak/mean velocities of microsaccades detected with the video tracker are consistent with previous studies showing larger peak/mean velocities of saccades (almost exclusively larger than 1 deg) with video-oculography than with search coil methods [27,31,32,34]. Only one study found no statistical differences between saccadic properties determined with a video-based infrared tracker and the search coil technique [30], but analyses were limited and their details scarce, thus precluding firm comparisons with the present study.

Some prior research has argued that video-oculography systems may produce less reliable measurements due to their low sampling rates [27]. This explanation is unlikely in light of the sampling rates in the present study (500 Hz) and in Kimmel et al.'s study [34] (1000 Hz).

Previous studies have also proposed that the contact lens used with the search coil technique applies an artificial load that slows down eye motion, or that contact lens slippage results in a low-pass filtered version of the true eye velocity [31]. The results of [34] (higher peak velocities with video tracking than with the search coil, in the same eye) argue against these possibilities, but we note that their primates had surgically-implanted search coils (thus precluding or at least diminishing slippage and load application). The present findings do not support the load theory either, because microsaccades measured by both systems in the same eye showed higher peak velocities with video tracking than with the search coil, in agreement with [33,34]. We cannot rule out lens slippage as a possibility, however.

An alternative explanation put forward by [34], and the most plausible one in our opinion, is that the different structures tracked by the two recording systems result in different saccadic peak velocities. The search coil tracks the globe’s position in space, whereas the video tracker tracks the position of the pupil—a space defined by the iris—, which is non-rigid. Furthermore the video tracker sees the pupil through the eye lens, which is also non-rigid. This theory (see [34] for more details) could explain the higher peak velocities for video tracking than for search coils found both in Kimmel et al's study and in the present research. In agreement with this possibility, a recent study showed that post-saccadic oscillations in pupil-based eye trackers reflect motion of the pupil inside the iris [60].

### Differences in drift speeds

Ocular drift was moderately, but significantly, correlated between the two systems, but drift speeds were higher for video tracker than for search coil data. Increased drift speeds as measured with the video tracker are probably due to increased noise compared to the search coil method (i.e. the speed of the data equals the speed of the real signal plus the speed of the noise). Variations in pupil size may also affect drift speeds measured with a video tracker [34,61], but not with a search coil system. Thus, task designs that require large changes in luminance would preclude video-based drift measurements, as noted by [34]. Constant luminance conditions, such as those in the present experiment, will produce moderate correlations between video tracker and search coil drift. However, when measuring ocular drift using a video tracker, one must carefully control not only luminance, but also arousal and any other variables known to influence pupil size [34,38].

A recent video tracking study found that drift speeds increased with time-on-task in human subjects [59]. Drift speeds observed at the beginning of the experiment were already higher than typically found with search coils or DPI trackers, in agreement with the present results. Despite higher-than-standard drift speeds, Di Stasi et al.'s conclusions (as well as those of studies conducted in comparable circumstances) remain valid, because drift was measured within a single system, and so any changes across experimental conditions are relative to that system. We also note that, whereas the drift speed estimate from video tracking may be dominated by noise (and thus higher than in reality) when drift speeds are low and noise is high, as drift speeds increase (for instance, with time-on-task), the drift speed estimate may be dominated by the true drift speed. Thus, one may detect relative changes in drift speed even in the presence of noise that would preclude a good estimate of the absolute value of (low) drift speed.

It is worth noting as well that, whereas true drift speeds may be lower than those measured with the video tracker, it could also be that true drift speeds are higher than those measured with the search coil technique.

### Noise

Noise complicates microsaccade detection and lowers the accuracy of microsaccade and drift properties measurements. Here, recording conditions were not ideal for video tracking (i.e. a contact lens was in the eye, the eye was anesthetized, and a wire was coming out the eye). Furthermore, the RMS was higher for the EyeLink data than the search coil data, suggesting higher levels of noise (mean RMS for this experiment: video tracker 7.1 ± 1.1 deg/s, search coil 4.0 ± 0.6 deg/s). Even so, there was substantial agreement between the two systems. Measurements in more ideal conditions might result in even closer agreement between EyeLink and search coil measurements. It must be noted that automated saccade detection algorithms may require different parameters for optimal saccadic detection under different levels of noise (i.e. as in [48]). Such optimal algorithm parameters are likely to differ for any two eye tracking systems (or even for the same system, with different subjects/conditions).

## Conclusions

We found substantial agreement between FEM measurements with EyeLink 1000 and the search coil technique. Whereas some of the measured microsaccade and drift properties did differ between the two systems, this should not matter for most studies using only one system, however (i.e. when measuring microsaccades and drift with a single system, any changes across experimental conditions will be relative to that system).

Microsaccade detecting ability is critical to FEM studies (as everything hinges on microsaccade detection, even the accuracy of drift measurements), and we show here that microsaccade detection is comparable for the EyeLink and the search coil system. Further, drift measurements with EyeLink were significantly correlated with those from the search coil. Thus, contemporary video tracking technology now approaches the search coil for measuring FEMs, while interfering less with natural vision and being less invasive.

These results are consistent with recent research showing comparable detection of saccades and fixation disparity with EyeLink 1000 and a DPI tracker [62] (DPI trackers, like search coil systems, are high performance eye trackers but also suffer from relatively invasive and complicated setups), in non-simultaneous recording conditions.

Previous to the 1990s, microsaccades were often defined as having amplitudes smaller than 12 arc min (i.e. 0.2 deg). This cut-off value originated in studies finding that the distribution of saccadic sizes during fixation declined sharply around 12 arc min ([63], but see [64]). Later studies found that microsaccade sizes frequently exceeded this value, however, often asymptoting around 1 deg [2,22,55,65]. The present results show that the increase in microsaccade sizes in the last ~15 years is not primarily due to the switch to video tracking methods, because the microsaccade magnitude distributions for both video tracker and search coil recordings found here extend well beyond 12 arc min.

## References

[1] Henderson JM. Human gaze control during real-world scene perception. Trends in Cognitive Sciences. 2003;7: 498–504. 10.1016/j.tics.2003.09.006 14585447

[2] Martinez-Conde S, Otero-Millan J, Macknik SL. The impact of microsaccades on vision: towards a unified theory of saccadic function. Nature Reviews Neuroscience. 2013;14: 83–96. 10.1038/nrn3405 23329159

[3] McCamy MB, Otero-Millan J, Di Stasi LL, Macknik SL, Martinez-Conde S. Highly informative natural scene regions increase microsaccade production during visual scanning. J Neurosci. 2014;34: 2956–2966. 10.1523/JNEUROSCI.4448-13.2014 24553936 PMC6608512

[4] Rayner K. Eye movements and attention in reading, scene perception, and visual search. The Quarterly Journal of Experimental Psychology. 2009;62: 1457–1506. 10.1080/17470210902816461 19449261

[5] Underwood G, Foulsham T, Humphrey K. Saliency and scan patterns in the inspection of real-world scenes: Eye movements during encoding and recognition. VISUAL COGNITION. 2009;17: 812Á834.

[6] Fahey MC, Cremer PD, Aw ST, Millist L, Todd MJ, White OB, et al Vestibular, saccadic and fixation abnormalities in genetically confirmed Friedreich ataxia. Brain. 2008;131: 1035–1045. 10.1093/brain/awm323 18238798

[7] Leigh RJ, Zee DS. The neurology of eye movements. Oxford Univ Press; 2006.

[8] Pinnock R, McGivern R, Forbes R, Gibson J. An exploration of ocular fixation in Parkinson’s disease, multiple system atrophy and progressive supranuclear palsy. J Neurol. 2010;257: 533–539. 10.1007/s00415-009-5356-3 19847469

[9] Serra A, Liao K, Martinez-Conde S, Optican LM, Leigh RJ. Suppression of saccadic intrusions in hereditary ataxia by memantine. Neurology. 2008;70: 810–2. 70/10/810 [pii] 10.1212/01.wnl.0000286952.01476.eb 18316692 PMC2740623

[10] Tatler BW, Hayhoe MM, Land MF, Ballard DH. Eye guidance in natural vision: Reinterpreting salience. Journal of Vision. 2011;11 10.1167/11.5.5 PMC3134223 21622729

[11] Tsotsos JK, Culhane SM, Kei Wai WY, Lai Y, Davis N, Nuflo F. Modeling visual attention via selective tuning. Artificial Intelligence. 1995;78: 507–545. 10.1016/0004-3702(95)00025-9

[12] Boraston Z, Blakemore S. The application of eye-tracking technology in the study of autism. The Journal of Physiology. 2007;581: 893–898. 10.1113/jphysiol.2007.133587 17430985 PMC2170849

[13] Gold JM, Fuller RL, Robinson BM, Braun EL, Luck SJ. Impaired top—down control of visual search in schizophrenia. Schizophrenia Research. 2007;94: 148–155. 10.1016/j.schres.2007.04.023 17544632 PMC1978542

[14] Martinez-Conde S. Fixational eye movements in normal and pathological vision Visual Perception—Fundamentals of Vision: Low and Mid-Level Processes in Perception. Elsevier; 2006 pp. 151–176. Available: http://www.sciencedirect.com.ezproxy1.lib.asu.edu/science/article/B7CV6-4M0C546-F/2/2321185cb4e661f44f2f859d714f5fbb 10.1016/S0079-6123(06)54008-7 17010709

[15] Otero-Millan J, Serra A, Leigh RJ, Troncoso XG, Macknik SL, Martinez-Conde S. Distinctive features of saccadic intrusions and microsaccades in progressive supranuclear palsy. J Neurosci. 2011;31: 4379–4387. 10.1523/JNEUROSCI.2600-10.2011 21430139 PMC3111217

[16] Di Stasi LL, Renner R, Catena A, Cañas JJ, Velichkovsky BM, Pannasch S. Towards a driver fatigue test based on the saccadic main sequence: A partial validation by subjective report data. Transportation Research Part C: Emerging Technologies. 2012;21: 122–133. 10.1016/j.trc.2011.07.002

[17] Inhoff AW, Gordon AM. Eye Movements and Eye-Hand Coordination during Typing. Current Directions in Psychological Science. 1997;6: 153–157.

[18] Land MF, Lee DN. Where we look when we steer. Nature. 1994;369: 742–744. 10.1038/369742a0 8008066

[19] Land MF. Vision, Eye Movements, and Natural Behavior. Visual Neuroscience. 2009;26: 51–62. 10.1017/S0952523808080899 19203425

[20] Carpenter RH. Movements of the Eyes. Pion; 1977.

[21] Ratliff F, Riggs LA. Involuntary motions of the eye during monocular fixation. Journal of Experimental Psychology. 1950;40: 687–701. 14803643 10.1037/h0057754

[22] Rolfs M. Microsaccades: Small steps on a long way. Vision Res. 2009;49: 2415–2441. 10.1016/j.visres.2009.08.010 19683016

[23] Steinman RM, Haddad GM, Skavenski AA, Wyman D. Miniature eye movement. Science. 1973;181: 810–819. 4198903 10.1126/science.181.4102.810

[24] Yarbus AL. Eye movements and vision. New York: Plenum press; 1967.

[25] Collewijn H, van der Mark F, Jansen TC. Precise recording of human eye movements. Vision Research. 1975;15: 447–IN5. 10.1016/0042-6989(75)90098-X 1136166

[26] Robinson DA. A method of measuring eye movements using a scleral coil in a magnetic field. IEEE Trans Biomed Eng. 1963;10: 137–145. 14121113 10.1109/tbmel.1963.4322822

[27] Houben MMJ, Goumans J, van der Steen J. Recording Three-Dimensional Eye Movements: Scleral Search Coils versus Video Oculography. Investigative Ophthalmology & Visual Science. 2006;47: 179–187. 10.1167/iovs.05-0234 16384960

[28] Imai T, Sekine K, Hattori K, Takeda N, Koizuka I, Nakamae K, et al Comparing the accuracy of video-oculography and the scleral search coil system in human eye movement analysis. Auris Nasus Larynx. 2005;32: 3–9. 10.1016/j.anl.2004.11.009 15882818

[29] McCamy MB, Macknik SL, Martinez-Conde S. Natural eye movements and vision The New Visual Neurosciences. Cambridge, MA, USA: MIT Press; 2014 pp. 849–863.

[30] Schmitt K-U, Muser MH, Lanz C, Walz F, Schwarz U. Comparing eye movements recorded by search coil and infrared eye tracking. J Clin Monit Comput. 2007;21: 49–53. 10.1007/s10877-006-9057-5 17120108

[31] Träisk F, Bolzani R, Ygge J. A comparison between the magnetic scleral search coil and infrared reflection methods for saccadic eye movement analysis. Graefe’s Arch Clin Exp Ophthalmol. 2005;243: 791–797. 10.1007/s00417-005-1148-3 15761761

[32] Van der Geest JN, Frens MA. Recording eye movements with video-oculography and scleral search coils: a direct comparison of two methods. Journal of Neuroscience Methods. 2002;114: 185–195. 10.1016/S0165-0270(01)00527-1 11856570

[33] Frens MA, Geest JNV der. Scleral Search Coils Influence Saccade Dynamics. J Neurophysiol. 2002;88: 692–698. 10.1152/jn.00457.2001 12163522

[34] Kimmel DL, Mammo D, Newsome WT. Tracking the eye non-invasively: simultaneous comparison of the scleral search coil and optical tracking techniques in the macaque monkey. Frontiers in Behavioral Neuroscience. 2012;6 10.3389/fnbeh.2012.00049 PMC3418577 22912608

[35] Cherici C, Kuang X, Poletti M, Rucci M. Precision of sustained fixation in trained and untrained observers. J Vis. 2012;12: 1–16. 10.1167/12.6.31 22728680 PMC3489479

[36] Haslwanter T, Clarke AH. Chapter 5—Eye movement measurement: electro-oculography and video-oculography Vertigo and Imbalance: Clinical Neurophysiologyof the Vestibular System. Elsevier; 2010 pp. 61–79. Available: http://www.sciencedirect.com/science/article/pii/S1567423110090052

[37] DiScenna AO, Das V, Zivotofsky AZ, Seidman SH, Leigh RJ. Evaluation of a video tracking device for measurement of horizontal and vertical eye rotations during locomotion. Journal of Neuroscience Methods. 1995;58: 89–94. 10.1016/0165-0270(94)00162-A 7475237

[38] Drewes J, Masson GS, Montagnini A. Shifts in reported gaze position due to changes in pupil size: ground truth and compensation Proceedings of the Symposium on Eye Tracking Research and Applications. New York, NY, USA: ACM; 2012 pp. 209–212. 10.1145/2168556.2168596

[39] Smeets JBJ, Hooge ITC. Nature of Variability in Saccades. J Neurophysiol. 2003;90: 12–20. 10.1152/jn.01075.2002 12611965

[40] Albano JE, Arzola Marrero J. Binocular interactions in rapid saccadic adaptation. Vision research. 1995;35: 3439–3450. 8560810 10.1016/0042-6989(95)00079-t

[41] Hopp JJ, Fuchs AF. The characteristics and neuronal substrate of saccadic eye movement plasticity. Progress in neurobiology. 2004;72: 27–53. 15019175 10.1016/j.pneurobio.2003.12.002

[42] Optican LM, Robinson DA. Cerebellar-dependent adaptive control of primate saccadic system. J Neurophysiol. 1980;44: 1058–1076. 7452323 10.1152/jn.1980.44.6.1058

[43] Snow R, Hore J, Vilis T. Adaptation of saccadic and vestibulo-ocular systems after extraocular muscle tenectomy. IOVS. 1985;26: 924–931. 3874190

[44] Krauskopf J, Cornsweet TN, Riggs LA. Analysis of eye movements during monocular and binocular fixation. J Opt Soc Am. 1960;50: 572 10.1364/JOSA.50.000572 14411808

[45] Martinez-Conde S, Macknik SL, Hubel DH. The role of fixational eye movements in visual perception. Nat Rev Neurosci. 2004;5: 229–240. 14976522 10.1038/nrn1348

[46] Eyelink Manual. 1.4.0 ed Mississauga, Ontario, Canada: SR Research Ltd.; 2008.

[47] Kapoula Z, Robinson DA, Hain TC. Motion of the eye immediately after a saccade. Exp Brain Res. 1986;61: 386–94. 3948945 10.1007/BF00239527

[48] Engbert R, Kliegl R. Microsaccades uncover the orientation of covert attention. Vision Res. 2003;43: 1035–1045. 10.1016/S0042-6989(03)00084-1 12676246

[49] Engbert R, Mergenthaler K. Microsaccades are triggered by low retinal image slip. Proc Natl Acad Sci USA. 2006;103: 7192–7197. 10.1073/pnas.0509557103 16632611 PMC1459039

[50] Engbert R. Microsaccades: A microcosm for research on oculomotor control, attention, and visual perception. Prog Brain Res. 2006;154: 177–192. 17010710 10.1016/S0079-6123(06)54009-9

[51] Laubrock J, Engbert R, Kliegl R. Microsaccade dynamics during covert attention. Vision Res. 2005;45: 721–730. 10.1016/j.visres.2004.09.029 15639499

[52] Rolfs M, Laubrock J, Kliegl R. Shortening and prolongation of saccade latencies following microsaccades. Exp Brain Res. 2006;169: 369–376. 10.1007/s00221-005-0148-1 16328308

[53] Møller F, Laursen M, Tygesen J, Sjølie A. Binocular quantification and characterization of microsaccades. Graefes Arch Clin Exp Ophthalmol. 2002;240: 765–770. 10.1007/s00417-002-0519-2 12271375

[54] Martinez-Conde S, Macknik SL, Troncoso XG, Dyar TA. Microsaccades counteract visual fading during fixation. Neuron. 2006;49: 297–305. 10.1016/j.neuron.2005.11.033 16423702

[55] Martinez-Conde S, Macknik SL, Troncoso XG, Hubel DH. Microsaccades: a neurophysiological analysis. Trends Neurosci. 2009;32: 463–475. 10.1016/j.tins.2009.05.006 19716186

[56] McCamy MB, Najafian Jazi A, Otero-Millan J, Macknik SL, Martinez-Conde S. The effects of fixation target size and luminance on microsaccades and square-wave jerks. PeerJ. 2013;1: e9 10.7717/peerj.9 23638403 PMC3628898

[57] McCamy MB, Collins N, Otero-Millan J, Al-Kalbani M, Macknik SL, Coakley D, et al Simultaneous recordings of ocular microtremor and microsaccades with a piezoelectric sensor and a video-oculography system. PeerJ. 2013;1: e14 10.7717/peerj.14 23638348 PMC3629042

[58] Troncoso XG, Macknik SL, Otero-Millan J, Martinez-Conde S. Microsaccades drive illusory motion in the Enigma illusion. Proc Natl Acad Sci USA. 2008;105: 16033–16038. 10.1073/pnas.0709389105 18843109 PMC2572936

[59] Di Stasi LL, McCamy MB, Catena A, Macknik SL, Cañas JJ, Martinez-Conde S. Microsaccade and drift dynamics reflect mental fatigue. European Journal of Neuroscience. 2013;38: 2389–2398. 10.1111/ejn.12248 23675850

[60] Nyström M, Hooge I, Holmqvist K. Post-saccadic oscillations in eye movement data recorded with pupil-based eye trackers reflect motion of the pupil inside the iris. Vision Research. 2013;92: 59–66. 10.1016/j.visres.2013.09.009 24096093

[61] Wyatt HJ. The human pupil and the use of video-based eyetrackers. Vision Research. 2010;50: 1982–1988. 10.1016/j.visres.2010.07.008 20638401 PMC2948855

[62] Kirkby JA, Blythe HI, Drieghe D, Benson V, Liversedge SP. Investigating eye movement acquisition and analysis technologies as a causal factor in differential prevalence of crossed and uncrossed fixation disparity during reading and dot scanning. Behav Res. 2013;45: 664–678. 10.3758/s13428-012-0301-2 23344736

[63] Collewijn H, Kowler E. The significance of microsaccades for vision and oculomotor control. Journal of Vision. 2008;8 10.1167/8.14.20 PMC3522523 19146321

[64] Fender DH. The function of eye movements in the visual process. University of Reading. 1956.

[65] Mergenthaler K, Engbert R. Microsaccades are different from saccades in scene perception. Experimental Brain Research. 2010;203: 753–757. 10.1007/s00221-010-2272-9 20467731

